# Race/Ethnicity–Specific Associations of Urinary Phthalates with Childhood Body Mass in a Nationally Representative Sample

**DOI:** 10.1289/ehp.1205526

**Published:** 2013-02-04

**Authors:** Leonardo Trasande, Teresa M. Attina, Sheela Sathyanarayana, Adam J. Spanier, Jan Blustein

**Affiliations:** 1 Department of Pediatrics,; 2 Department of Environmental Medicine, and; 3 Department of Population Health, New York University School of Medicine, New York, New York, USA; 4 New York University Wagner School of Public Service, New York, New York, USA; 5 New York University Steinhardt School of Culture, Education, and Human Development, New York, New York, USA; 6 Seattle Children’s Research Institute, Department of Pediatrics, University of Washington, Seattle, Washington, USA; 7 Department of Pediatrics, Penn State University, Hershey, Pennsylvania, USA

**Keywords:** body mass, obesity, phthalates, racial/ethnic disparities

## Abstract

Background: Phthalates have antiandrogenic effects and may disrupt lipid and carbohydrate metabolism. Racial/ethnic subpopulations have been documented to have varying urinary phthalate concentrations and prevalences of childhood obesity.

Objective: We examined associations between urinary phthalate metabolites and body mass outcomes in a nationally representative sample of U.S. children and adolescents.

Methods: We performed stratified and whole-sample cross-sectional analyses of 2,884 children 6–19 years of age who participated in the 2003–2008 National Health and Nutrition Examination Survey. Multivariable linear and logistic analyses of body mass index *z*-score, overweight, and obesity were performed against molar concentrations of low-molecular-weight (LMW), high-molecular-weight (HMW), and di-2-ethylhexylphthalate (DEHP) metabolites, controlling for sex, television watching, caregiver education, caloric intake, poverty–income ratio, race/ethnicity, serum cotinine, and age group. We used sensitivity analysis to examine robustness of results to removing sample weighting, normalizing phthalate concentrations for molecular weight, and examining different dietary intake covariates.

Results: In stratified, multivariable models, each log unit (roughly 3-fold) increase in LMW metabolites was associated with 21% and 22% increases in odds (95% CI: 1.05–1.39 and 1.07–1.39, respectively) of overweight and obesity, and a 0.090-SD unit increase in BMI *z*-score (95% CI: 0.003–0.18), among non-Hispanic blacks. Significant associations were not identified in any other racial/ethnic subgroup or in the study sample as a whole after controlling for potential confounders, associations were not significant for HMW or DEHP metabolites, and results did not change substantially with sensitivity analysis.

Conclusions: We identified a race/ethnicity–specific association of phthalates with childhood obesity in a nationally representative sample. Further study is needed to corroborate the association and evaluate genetic/epigenomic predisposition and/or increased phthalate exposure as possible explanations for differences among racial/ethnic subgroups.

Phthalates are diesters of phthalic acid that are used to produce an array of consumer products and have been associated with abnormalities in end points related to endocrine processes, including reproductive and neurodevelopmental outcomes (Bornehag and Nanberg 2010; Colon et al. 2000; Engel et al. 2010; Miodovnik et al. 2011; Sathyanarayana 2008; Swan 2008; Swan et al. 2005). A causal role for phthalates in obesity is biologically plausible, based in part on evidence of high-molecular-weight (HMW) phthalate interactions with three peroxisome proliferator–activated receptors (PPARs) that are members of the nuclear receptor superfamily (Desvergne et al. 2009). PPARs are involved in lipid and carbohydrate metabolism; therefore, their activation may represent a mechanism by which phthalates could produce metabolic derangements that might contribute to obesity (Grün and Blumberg 2007).

Three analyses have examined urinary phthalates and obesity in humans to date. The first reported that urinary phthalates were associated with abdominal obesity and insulin resistance in adults in the 1999–2002 U.S. National Health and Nutrition Examination Survey (NHANES) (Stahlhut et al. 2007). In contrast, quartiled urinary phthalate concentrations in 1,209 children and adolescents in the 1999–2002 NHANES were not associated with unstandardized measurements of body mass index (BMI) or waist circumference (WC) (Hatch et al. 2008), although patterns of association varied by age and sex. Finally, in a population of largely Latino, New York City children (Teitelbaum et al. 2012), urinary phthalates measured in 6- to 8-year-olds were not associated with BMI and WC 1 year later in the population as a whole, but body mass measures were positively associated with log-transformed phthalate metabolites for monoethyl phthalate (MEP) and with the sum of LMW phthalate metabolites [MEP, mono-*n*-butyl-phthalate (MBP), monoisobutyl phthalate (MiBP), and mono(3-carboxypropyl) phthalate (MCPP)] among overweight children.

Racial/ethnic differences in creatinine excretion are well documented (Barr et al. 2005), as are differences in urinary phthalate concentrations (Silva et al. 2004; Wolff et al. 2007). The prevalence of obesity also varies by race/ethnicity (Freedman et al. 2006; Ogden et al. 2012; Strauss and Pollack 2001), raising the possibility that racial/ethnic differences in phthalate exposures may partly explain racial/ethnic differences in obesity. Yet despite the possibility that differences in obesogenic exposures or genetic/epigenomic predisposition may contribute to differences in obesity, and the potentially substantial implications this would have for targeting prevention efforts, few studies have examined racial/ethnic differences in environmental risk factors for childhood obesity.

We therefore analyzed data from the 2003–2008 NHANES to study associations of urinary phthalate metabolites with body mass and examine differences according to race/ethnicity and other factors.

## Methods

*Data source and sample.* NHANES is a continuous, multicomponent, nationally representative survey of the noninstitutionalized U.S. population administered by the National Centers for Health Statistics (NCHS) of the Centers for Disease Control and Prevention (CDC 2012b). We used data from the questionnaire, laboratory, diet, and physical examination components in the present analysis, for which data are available in biennial groupings. Our analytic sample comprised 2,884 nonpregnant participants 6–19 years of age with urinary phthalate measurements. NHANES is approved by the NCHS Research Ethics Review Board, and written informed consent and child assent (as appropriate) was obtained from participants. The NYU School of Medicine Institutional Review Board exempted the present study from review because it is based on previously collected and deidentified data.

*Measurement of urinary phthalates*. Phthalates were measured in a spot urine sample collected from a randomly selected subsample of NHANES participants using high-performance liquid chromatography and tandem mass spectroscopy, as previously described (Silva et al. 2004). Phthalate concentrations below the level of detection [5.1% for mono(2-ethylhexyl) phthalate (MEHP), < 1% for all other metabolites studied] were assigned the limit of detection divided by the square root of 2, as recommended by NHANES. All models included urinary creatinine to adjust for urine dilution, following usual practice (Barr et al. 2005; Stahlhut et al. 2009).

We grouped biomarkers according to use. Low-molecular-weight (LMW) phthalates (diethyl phthalate, di-*n*-butyl phthalate, di-*n*-octylphthalate and di-*n*-isobutyl phthalate) are predominantly used in shampoos, cosmetics, lotions and other personal care products to preserve scent (Hauser and Calafat 2005; Sathyanarayana 2008; Sathyanarayana et al. 2008), whereas HMW phthalates [di(2-ethylhexyl) phthalate (DEHP), di-*n*-octyl phthalate and butylbenzyl phthalate) are used to produce vinyl plastic for flooring, clear food wrap, intravenous tubing, and other products (Schettler 2006). DEHP is of particular interest because industrial processes to produce food frequently use plastic products containing DEHP (Fromme et al. 2007; Wormuth et al. 2006).

We expressed the concentration of LMW phthalate metabolites as the sum of molar concentrations of MEP, MBP, and MiBP. The concentration of HMW metabolites was calculated as the sum of molarities of mono(2-ethyl-5-carboxypentyl) phthalate (MECPP), MCPP, mono(2-ethyl-5-hydroxyhexyl) phthalate (MEHHP), mono(2-ethyl-5-oxohexyl) phthalate (MEOHP), MEHP, and monobenzyl phthalate (MBzP). Finally, we calculated the DEHP metabolite concentration by adding molarities of MEHP, MECPP, MEHHP, and MEOHP.

Our primary exposure variables were the natural log-transformed total molar concentrations of LMW, HMW, and DEHP metabolites. In addition, we estimated associations with metabolite groups categorized into tertiles, and with selected individual phthalate metabolites.

*Body mass outcomes*. In the NHANES, trained health technicians assessed body measurements, following published, standardized measurement procedures (Lohman et al. 1998). BMI was calculated by dividing the weight in kilograms by the height in meters squared, and *z*-scores were derived from 2000 CDC reference growth curves (Kuczmarski et al. 2002)using the *zanthro* command in Stata 12.0 (StataCorp., College Station, TX). Overweight and obese were classified as BMI *z*-score ≥ 85th percentile for age and sex and ≥ 95th percentile, respectively (Grummer-Strawn et al. 2010; Ogden et al. 2002). Study outcomes were obesity (BMI *z*-score ≥ 95th percentile vs. < 95th percentile), overweight (BMI *z*-score ≥ 85th percentile vs. < 85th percentile), and BMI *z*-score (as continuous variable).

*Potential confounders and other covariates*. Trained interviewers fluent in Spanish and English elicited two total 24-hr dietary recalls using standard measuring guides to assist reporting of volumes and dimensions of food items, and responses were converted to energy and nutrients by appropriate nutritional software (CDC 2012a). We used the first of the two 24-hour recalls in the present analysis. Because the measurement of physical activity changed during the study period, we were unable to categorize physical activity into low, medium, and high groups normally used to derive caloric needs based on age- and sex-specific U.S. Department of Agriculture (USDA) guidelines (USDA 2010). Therefore, as a conservative measure, we categorized participants into “normal” or “excessive” caloric intake groups based upon daily caloric guidelines for high physical activity children, recognizing that this probably underestimates the proportion who exceeded USDA calorie intake guidelines. We dichotomized self-reported television watching as < 2 or ≥ 2 hr/day, in light of previous associations with obesity (American Academy of Pediatrics Committee on Public Education 2001), and associations with urinary phthalates in our study sample. Because exposure to tobacco smoke is a risk factor for metabolic syndrome in adolescence (Weitzman et al. 2005), and because serum cotinine was positively associated with urinary phthalate metabolites in our study population, we included serum cotinine in multivariable models categorized as low (< 0.015 ng/mL), medium (0.015–2 ng/mL), or high (≥ 2 ng/mL).

Race/ethnicity was categorized as Mexican American, other Hispanic, non-Hispanic white, non-Hispanic black, and other, based on self-report by 17- to 19-year-olds and caregiver report for 6- to 16-year-olds. Caregiver education was categorized as less than 9th grade, 9th–12th grade, high school/graduate equivalency diploma, some college, and college or greater. Poverty–income ratio (annual household income/poverty level) was categorized into quartiles. Age was categorized as 6–11 or 12–19 years. To maximize sample sizes in multivariable analyses, we included “missing” categories for all potential confounders. Television watching was missing for 24.4%, and serum cotinine was missing for 9.6%. Otherwise, < 5% of values were missing for any covariate. Recognizing concerns raised about potential bias due to the use of missing data categories in regression models (Jones 1996), we repeated our main model as a complete case analysis, omitting participants with missing values for any of the covariates.

*Statistical analysis*. We conducted univariable, bivariable, and multivariable analyses using statistical techniques that account for the complex survey sampling design, using Stata 12.0, and following NCHS guidelines (CDC 2012b). We used multivariable linear regression analysis to model BMI *z*-score, and logistic regression to model categorical overweight and obesity in separate models.

We used log-transformed LMW, HMW, and DEHP urinary metabolite concentrations in our analyses to account for skew in the distribution of urinary phthalates. We performed separate univariate regressions of each exposure against BMI *z*-score, overweight, obesity, and covariates. We adjusted all multivariable models for urinary creatinine (model A). Next we added demographic and exposure characteristics (race/ethnicity, age, caregiver education, poverty–income ratio, sex, serum cotinine) (model B), and then lifestyle characteristics (measures of caloric intake, television watching) (model C).

We also developed univariate and multivariable regression models of the phthalate-obesity association stratified by sex, for which differences in urinary phthalates have also been noted, age (6–11 or 12–19 years), poverty–income ratio (< 1.6 or ≥ 1.6), cotinine level (< 2.0 or ≥ 2.0 ng/mL), parent education (no college or at least some college), caloric intake (excessive or appropriate), and television watching (< 2 or ≥ 2 hr/day). In addition, we stratified on race/ethnicity, classified as non-Hispanic black, Hispanic (Mexican-American and other Hispanic combined), and non-Hispanic white to maintain large stratum-specific samples. As a test of robustness, we estimated associations according to race/ethnicity by modeling product interaction terms for the exposure and potential modifier, in addition to lower-order terms and covariates, in whole-sample regression models controlling for all covariates. These models did not combine Hispanics into one group, maintaining the subgroupings of other Hispanics and Mexican Americans used by NHANES. In secondary analyses, we analyzed individual phthalate metabolites according to race/ethnicity.

To ensure that our results were not an artifact of statistical weighting, we also repeated our analysis of race/ethnicity–stratified models in unweighted modeling. We also reprised our models substituting continuous kilocalories in lieu of categorized excessive/appropriate caloric intake for age and sex. Finally, we repeated our analyses, substituting continuous for categorized age, and recalculated LMW, HMW, and DEHP concentrations by weighting molar concentrations using each metabolite’s molecular weight, following published practice (Teitelbaum et al. 2012).

## Results

Geometric mean concentrations of LMW phthalates metabolites were higher among girls than boys and among adolescents than children 6–11 years of age (Table 1). HMW phthalates were also significantly higher among adolescents. Non-Hispanic blacks had higher concentrations of all metabolites than Mexican Americans, whereas non-Hispanic whites had lower concentrations of urinary LMW metabolites and higher concentrations of HMW and DEHP metabolites. The highest half of the income distribution had lower LMW phthalates than the lower half. Children with medium and high urinary cotinine also had steadily higher LMW concentrations than children with low concentrations. Excessive caloric intake was associated with significantly lower LMW and significantly higher HMW and DEHP concentrations, whereas ≥ 2 hours/day of television watching was associated with higher LMW concentrations. Anthropometric measurements were not available in 22 participants, leaving a final sample size of 2,862 for regression analysis of biomarker-outcome associations.

**Table 1:** Geometric mean (GM) urinary molar concentration of metabolites of the three phthalate categories, by sample characteristics (*n* = 2,884).

Characteristic	Total n (%)a	GM urinary LMW metabolite (µM)	p-Valueb	GM urinary HMW metabolite (µM)	p-Valueb	GM urinary DEHP metabolite (µM)	p-Valueb
Sex							
Male	1,487 (51.6)	0.593	Reference	0.525	Reference	0.358	Reference
Female	1,397 (48.5)	0.680	0.004	0.516	0.767	0.360	0.929
Age (years)							
6–11	1,087 (42.2)	0.546	Reference	0.559	Reference	0.381	Reference
12–19	1,797 (57.8)	0.894	< 0.001	0.480	0.025	0.347	0.187
Race/ethnicity							
Hispanic–Mexican American	863 (12.4)	0.793	Reference	0.433	Reference	0.308	Reference
Hispanic–other Hispanic	175 (5.6)	1.108	0.006	0.539	0.110	0.383	0.111
Non-Hispanic white	792 (61.2)	0.635	0.001	0.521	0.008	0.365	0.020
Non-Hispanic black	926 (15.0)	1.151	< 0.001	0.565	< 0.001	0.400	< 0.001
Other	128 (5.9)	0.500	< 0.003	0.455	0.715	0.329	0.647
Poverty–income ratio							
First quartile (< 0.83)	702 (17.6)	0.850	Reference	0.566	Reference	0.405	Reference
Second quartile (0.83 to 1.59)	702 (18.2)	0.851	0.990	0.532	0.493	0.369	0.339
Third quartile (1.60 to 3.09)	666 (25.0)	0.695	0.027	0.493	0.120	0.343	0.083
Fourth quartile (at least 3.1)	679 (35.5)	0.646	0.001	0.496	0.160	0.353	0.177
Missing	135 (3.7)	0.656	0.171	0.463	0.260	0.331	0.268
Parent/caregiver education							
< 9th grade	356 (6.8)	0.793	Reference	0.485	Reference	0.344	Reference
9th–12th grade	504 (12.5)	0.972	0.066	0.565	0.149	0.390	0.258
High school or GED	708 (24.4)	0.789	0.965	0.505	0.707	0.348	0.919
Some college	797 (31.6)	0.733	0.494	0.533	0.396	0.377	0.451
College or greater	396 (21.4)	0.548	0.006	0.492	0.886	0.357	0.747
Missing	123 (3.3)	0.623	0.191	0.393	0.227	0.280	0.281
Serum cotinine							
< 0.015 ng/mL	446 (15.6)	0.624	Reference	0.446	Reference	0.316	Reference
0.015–1.9 ng/mL	1,717 (57.8)	0.730	0.047	0.522	0.097	0.368	0.137
At least 2.0 ng/mL	394 (14.4)	0.948	< 0.001	0.515	0.240	0.356	0.364
Missing	327 (12.2)	0.628	0.972	0.556	0.084	0.399	0.080
Television watching							
< 2 hr/day	1,096 (28.7)	0.644	Reference	0.506	Reference	0.361	Reference
≥ 2 hr/day	1,294 (49.5)	0.760	0.015	0.545	0.246	0.376	0.551
Missing	494 (21.8)	0.768	0.031	0.453	0.363	0.330	0.479
Caloric intake compared with needs in active child of same age and sex							
Appropriate	1,924 (67.5)	0.744	Reference	0.489	Reference	0.344	Reference
Excessive	819 (28.1)	0.691	0.011	0.574	0.001	0.408	0.011
Missing	141 (4.4)	0.685	0.848	0.506	0.342	0.360	0.805
Obesity status							
Not obese	2,284 (83.2)	0.701	Reference	0.504	Reference	0.356	Reference
Obese	578 (16.8)	0.855	0.825	0.557	0.236	0.390	0.310
aAll percentages are weighted using population weights for the sample in which phthalates were measured. Total number of subjects from some variables (e.g., obesity status) do not total to 2,884 due to missing data. bDerived using regression of log-molar concentration of urinary phthalate against characteristic. Geometric mean urinary phthalate represents retransformed mean from log base.							

Table 2 presents associations of LMW, HMW and DEHP metabolites in relationship to body mass outcomes in the entire study population. Controlling for urinary creatinine (model A), a 1-unit increase in log-LMW metabolites was associated with a 0.07-SD unit increase in BMI *z*-score (95% CI: 0.02, 0.13; *p* = 0.012), and with a nonsignificant increase in obesity [odds ratio (OR) = 1.12; 95% CI: 0.99, 1.25; *p* = 0.063]. However, associations were consistent with the null after further adjustment for confounding, and neither HMW nor DEHP metabolites were significantly associated with body mass outcomes in any regression model.

**Table 2:** Associations between natural log-transformed urinary phthalate metabolite concentrations and BMI z-score [β coefficient (95% CI)], overweight, and obesity [OR (95% CI)] (n = 2,862).

Outcome	Model A	Model B	Model C
BMI z-score increment
LMW metabolites	0.07 (0.02, 0.13)*	0.03 (–0.03, 0.09)	0.03 (–0.03, 0.09)
HMW metabolites	–0.003 (–0.05, 0.05)	–0.01 (–0.06, 0.04)	–0.01 (–0.06, 0.04)
DEHP metabolites	–0.01 (–0.06, 0.04)	–0.02 (–0.07, 0.03)	–0.01 (–0.06, 0.03)
Overweight OR
LMW metabolites	1.08 (0.97, 1.19)	1.01 (0.90, 1.14)	1.01 (0.90, 1.13)
HMW metabolites	0.97 (0.86, 1.10)	0.96 (0.85, 1.08)	0.96 (0.86, 1.09)
DEHP metabolites	0.95 (0.84, 1.08)	0.95 (0.84, 1.06)	0.95 (0.85, 1.07)
Obesity OR
LMW metabolites	1.12 (0.99, 1.25)	1.02 (0.89, 1.16)	1.02 (0.90, 1.17)
HMW metabolites	1.02 (0.89, 1.17)	1.03 (0.88, 1.20)	1.04 (0.89, 1.22)
DEHP metabolites	1.01 (0.88, 1.16)	1.02 (0.88, 1.19)	1.04 (0.89, 1.20)
Model A adjusts only for urinary creatinine; model B also includes sex, poverty–income ratio, parental education, serum cotinine, age, and race/ethnicity category; model C includes model B covariates plus caloric intake and television watching. All associations are with the natural log of the phthalate metabolite concentration. Results represent increase in odds of BMI z-score per unit log increase in phthalate metabolite. *p < 0.05.			

LMW metabolites were significantly associated with BMI *z*-score and overweight in children with high caregiver education and appropriate caloric intake subpopulations in models adjusted for urinary creatinine, but associations were consistent with the null in both population subgroups after adjustment for other covariates (data not shown). LMW metabolites were not associated with the outcomes when stratified according to age group, sex, poverty–income ratio, television watching, or serum cotinine, and there were no significant associations of HMW or DEHP metabolites with body mass outcomes in any models stratified on these characteristics (data not shown).

Table 3 presents full, stratified multivariable models (model C) for associations of LMW, HMW, and DEHP metabolites with body mass outcomes within each of the major racial/ethnic groups studied within NHANES (Hispanics as a combined group, non-Hispanic whites, and non-Hispanic blacks). Within Hispanic and white populations, no significant associations were identified. However, LMW metabolites were significantly associated with all three of the body mass outcomes among non-Hispanic blacks, such that each log unit increase in LMW metabolites was associated with a 0.090-SD unit increase in BMI *z*-score (95% CI: 0.003, 0.18; *p* = 0.043) and ORs of 1.21 (95% CI: 1.05, 1.39; *p* = 0.008) and 1.22 (95% CI: 1.07, 1.39; *p* = 0.003) for overweight and obese, respectively.

**Table 3:** Associations between natural log-transformed urinary phthalate metabolite concentrations and BMI z-score [β coefficient (95% CI)], overweight, and obesity [OR (95% CI)] stratified by racial/ethnic group.

Outcome	Hispanic (n = 1026)	White (n = 792)	Black (n = 918)
BMI z-score increment
LMW metabolites	–0.04 (–0.15, 0.06)	0.02 (–0.08, 0.12)	0.09 (0.003, 0.18)*
HMW metabolites	0.02 (–0.06, 0.09)	–0.01 (–0.09, 0.06)	0.02 (–0.06, 0.10)
DEHP metabolites	0.02 (–0.04, 0.09)	–0.03 (–0.09, 0.04)	0.01 (–0.07, 0.08)
Overweight OR			
LMW metabolites	0.88 (0.72, 1.08)	0.97 (0.78, 1.22)	1.21 (1.05, 1.39)**
HMW metabolites	0.89 (0.73, 1.08)	0.98 (0.82, 1.18)	1.05 (0.89, 1.24)
DEHP metabolites	0.94 (0.79, 1.12)	0.95 (0.79, 1.13)	1.03 (0.88, 1.21)
Obesity OR			
LMW metabolites	0.97 (0.83, 1.14)	0.94 (0.69, 1.29)	1.22 (1.07, 1.39)**
HMW metabolites	1.10 (0.93, 1.29)	1.00 (0.76, 1.32)	1.13 (0.97, 1.32)
DEHP metabolites	1.13 (0.98, 1.30)	0.99 (0.76, 1.28)	1.11 (0.96, 1.29)
All models include sex, caloric intake, television watching, poverty–income ratio, parental education, serum cotinine, urinary creatinine, and age categories. Hispanics include both Mexican Americans and other Hispanics. *p < 0.05. **p < 0.01.			

Estimates from fully adjusted models that included race/ethnicity–LMW interaction terms also indicated significant differences in associations in the non-Hispanic black subgroup for obesity (OR = 1.14; 95% CI: 1.004, 1.29; *p* = 0.044) and BMI *z*-score (0.081-SD unit increase, 95% CI: 0.01, 0.15; *p* = 0.027) and a near significant association with overweight (OR = 1.13; 95% CI: 0.998, 1.27; *p* = 0.053), consistent with findings from separate models for non-Hispanic blacks. Estimates for other race/ethnicity groups based on the interaction models also were consistent with stratum-specific models.

Different sample weighting produced similar point estimates (0.07-SD unit increase in BMI *z*-score, OR = 1.29 for obesity, and OR = 1.13 for overweight among non-Hispanic blacks), confirming the robustness of the race/ethnicity-stratified multivariable results. Complete case analysis (*n* = 1,892) produced more modest point estimates (0.05-SD unit increase in BMI *z*-score, OR = 1.11 for obesity, and OR = 1.05 for overweight among non-Hispanic blacks). When we substituted continuous kilocalorie intake for the categorical normal/excessive caloric intake variable, the point estimates were also similar (0.09-SD unit increase in BMI *z*-score, OR = 1.16 for obesity, and OR = 1.14 for overweight among non-Hispanic blacks). Substitution of molecular weighted-concentrations also failed to significantly change the point estimates (data not shown). Substitution of continuous for categorized age also did not distort the point estimates.

Associations between individual log-molar LMW metabolites (MEP, MBP, and MiBP) and body mass outcomes among non-Hispanic blacks are shown in Table 4. A log-increase in urinary MEP was significantly associated with obesity (OR = 1.19; 95% CI: 1.05, 1.35; *p* = 0.007), overweight (OR = 1.18; 95% CI: 1.04, 1.34; *p* = 0.011), and BMI *z*-score (0.08-SD unit increase, 95% CI: 0.01, 0.16; *p* = 0.035). In addition, a log-increase in MBP was associated with obesity (OR = 1.21; 95% CI: 1.00, 1.45, *p* = 0.044). Nonsignificant associations were estimated for MiBP and overweight (OR = 1.16; 95% CI: 0.99, 1.37; *p* = 0.072), and BMI *z*-score (0.08-SD unit increase; 95% CI: –0.01, 0.17; *p* = 0.089). Estimates from full multivariable models that included race/ethnicity interaction terms also indicated significant associations between MEP and obesity (OR = 1.12; 95% CI: 1.001, 1.26), overweight (OR = 1.12; 95% CI: 1.01, 1.24; *p* = 0.026), and BMI *z*-score (0.08-SD unit increase; 95% CI: 0.02, 0.14; *p* = 0.008) among non-Hispanic blacks.

**Table 4:** Associations of individual natural log-transformed urinary LMW phthalate metabolites and BMI z-score [β coefficient (95% CI)], overweight, and obesity [OR (95% CI)] among non-Hispanic blacks.

Outcome	Estimate (95% CI)
BMI z-score
MEP	0.08 (0.01, 0.16)*
MBP	0.08 (–0.02, 0.18)
MiBP	0.08 (–0.01, 0.17)
Overweight
MEP	1.18 (1.04, 1.34)*
MBP	1.11 (0.93, 1.33)
MiBP	1.16 (0.99, 1.37)
Obesity
MEP	1.19 (1.05, 1.35)**
MBP	1.21 (1.00, 1.45)*
MiBP	1.17 (0.97, 1.41)
All models were controlled for sex, caloric intake, television watching, poverty–income ratio, parental education, serum cotinine, urinary creatinine, and age categories. *p < 0.05. **p < 0.01.	

## Discussion

We have found associations of urinary phthalate biomarkers with increased body mass among non-Hispanic black children in a nationally representative sample. Translated for the average 12-year-old (with weight 40 kg and height 150 cm), an increase in LMW phthalate concentration from the 25th to the 75th percentile among non-Hispanic blacks (a 1.5 log-unit difference) would be associated with a 0.8-kg increase in body weight, a 1.5% increment in prevalence of obesity, and a 3.3% increment in prevalence of overweight.

Although our results differ from those for children and adolescents in NHANES 1999–2002 (Hatch et al. 2008), our findings are consistent with evidence from a convenience sample of New York City children (Teitelbaum et al. 2012). No specific mechanism has been put forth for LMW phthalates, but other studies have identified similar associations (Stahlhut et al. 2007; Teitelbaum et al. 2012), suggesting the need for further studies of possible mechanisms for LMW phthalates to influence body mass in early life. And although the New York City study estimated associations of urinary phthalates with body mass 1 year later, our design was cross-sectional. The association we find is subject to concerns about reverse causality—for example, obese children may eat more packaged foods that contain phthalates, and thus have higher urinary levels. However, in our data, HMW and DEHP metabolites were not associated with body mass outcomes, whereas LMW metabolites, which derive from application of cosmetic and other personal care products, were. Our models included a variety of demographic, exposure, and lifestyle variables, thus providing more convincing evidence for nonspuriousness of the association. However, many other environmental exposures were not measured, including bisphenol A (BPA), which has been associated with childhood obesity in NHANES 2003–2008 (Trasande et al. 2012). Because children are likely to be exposed to multiple environmental chemicals, and because these chemical exposures may be correlated with each other, it is challenging to isolate associations. Simultaneous analysis of BPA with phthalates was not possible due to their collection in different subsamples of NHANES 2003–2004.

Phthalates are a heterogeneous group. Using best evidence from prior practice, we aggregated biomarkers into three groups (LMW, HMW, and DEHP metabolites). Our strategy was to examine associations within the three groups and then to look for impacts within population strata, given prior reports of differences in exposure across groups. In our analysis, then, associations were subject to multiple comparisons. It is possible that associations between body mass outcomes and LMW exposure among non-Hispanic blacks were driven by chance or uncontrolled bias within this population subgroup. Animal studies support an association between phthalates and obesity, but the association is most plausible for metabolites of DEHP, rather than the LMW metabolites reported here. LMW phthalates have been linked to obesity in other human studies (Stahlhut et al. 2007; Teitelbaum et al. 2012). One explanation for the consistent LMW association across this study and the other two human studies is that MBP, an LMW metabolite with potential antiandrogenic effects, is metabolized readily to MEP (Committee on the Health Risks of Phthalates–National Research Council 2008). Moreover, we found associations with all three outcomes among non-Hispanic blacks, and the association was robust to several sensitivity analyses.

It is unclear why the race/ethnicity–specific association would occur. One possibility is that different racial/ethnic groups use phthalate-containing shampoos and lotions differently, or use products containing different mixes of LMW compounds. Supporting this hypothesis is a study in a population of Mexican women, in which the number of personal care products used was positively correlated with urinary MEP, and increases in multiple LMW phthalates were associated with use of multiple types of personal care products (Romero-Franco et al. 2011). Higher concentrations among non-Hispanic blacks such as those identified in cross-sectional NHANES surveys may represent the ranges in which LMW phthalate effects on body mass occur.

Relationships between phthalate (including DEHP) intake and urinary metabolites are complex (Frederiksen et al. 2007). We know of no pharmacokinetic studies in children or adolescents, and population studies are limited in their capacity to evaluate exposure-excretion relationships. In animal studies, MBP is more plausibly associated with antiandrogenic activity and body mass effects than MEP (Committee on the Health Risks of Phthalates–National Research Council 2008). Indeed, log-transformed MEP and MBP were moderately correlated (*r* = 0.40) in our sample. Di-*n*-butyl phthalate (DBP; typically metabolized to MBP) and diethyl phthalate (typically metabolized to MEP) may coexist in products, and MBP metabolism to MEP (Frederiksen et al. 2007) may explain the association of body mass with MEP identified in our study and the one from a New York City sample (Teitelbaum et al. 2012).

Other authors have derived indices of LMW, HMW, and DEHP metabolite exposures by weighting concentrations of individual metabolites according to the molecular weight of individual phthalates, under the presumptive hypothesis that molecular weight is related to potency (Teitelbaum et al. 2012; Wolff et al. 2007). We followed this approach initially, and we found nearly identical results. Absent a potency-weighted scaling that accounts for androgen antagonism, PPAR activation, and other pathways by which phthalates might affect body mass, we chose to use unweighted, aggregate molar concentrations and present alternative weighting of concentrations as a sensitivity analysis.

Phthalate exposure was measured at one time point for this analysis, and monoesters of phthalates typically have half-lives of 12–48 hr (Hoppin et al. 2002), yet fat deposition of phthalates may also lengthen the half-life and may contribute both to obesity and increments in urinary phthalate metabolites (Mes et al. 1974). Urinary phthalates do represent current exposure better than chronic exposure. Yet one study has suggested that a single urine sample may classify exposure over the previous 3 months with higher sensitivity (63%) and specificity (87%) for MEP (an LMW metabolite), than HMW and DEHP metabolites (Hauser et al. 2004). This suggests that a single urine sample is not poor at estimating urine MEP chronically, and MEP accounted for the vast majority of the LMW metabolites in our study population. Even if current urinary phthalates are weak indices of chronic exposure, our estimates of association should be biased toward the null for dichotomous outcomes (Carroll 1998; Fleiss and Shrout 1977; Fuller 1987).

In contrast with adult studies (Stahlhut et al. 2007), we did not observe differences according to sex, thus suggesting a mechanism that does not involve antiandrogenic effects. The linear relationship with the logarithm suggests increasing incremental effects at lower ranges of exposure, consistent with studies of lead (Canfield et al. 2003; Lanphear et al. 2000) and methylmercury (Grandjean et al. 1997) exposures.

## Conclusion

We identified a race/ethnicity–specific association of phthalates with childhood obesity in a nationally representative sample. Further study is needed to corroborate the association and evaluate genetic/epigenomic predisposition and increased phthalate exposure as possible explanations for differences among racial/ethnic subgroups.

## References

[r1] American Academy of Pediatrics Committee on Public Education (2001). Children, adolescents, and television.. Pediatrics.

[r2] Barr DB, Wilder LC, Caudill SP, Gonzalez AJ, Needham LL, Pirkle JL (2005). Urinary creatinine concentrations in the U.S. population: implications for urinary biologic monitoring measurements.. Environ Health Perspect.

[r3] Bornehag CG, Nanberg E (2010). Phthalate exposure and asthma in children.. Int J Androl.

[r4] Canfield RL, Henderson CR, Cory-Slechta DA, Cox C, Jusko TA, Lanphear BP (2003). Intellectual impairment in children with blood lead concentrations below 10 µg per deciliter.. N Engl J Med.

[r5] Carroll RJ (1998). Measurement error in epidemiologic studies.

[r6] CDC (Centers for Disease Control and Prevention) (2012a). Measuring Guides for the Dietary Recall Interview.. http://www.cdc.gov/nchs/nhanes/measuring_guides_dri/measuringguides.htm.

[r7] CDC (Centers for Disease Control and Prevention) (2012b). Overview of NHANES Survey Design and Weights.. http://www.cdc.gov/nchs/tutorials/dietary/surveyorientation/surveydesign/intro.htm.

[r8] Colon I, Caro D, Bourdony CJ, Rosario O (2000). Identification of phthalate esters in the serum of young Puerto Rican girls with premature breast development.. Environ Health Perspect.

[r9] Committee on the Health Risks of Phthalates–National Research Council (2008). Phthalates and Cumulative Risk Assessment: The Task Ahead.

[r10] Desvergne B, Feige JN, Casals-Casas C (2009). PPAR-mediated activity of phthalates: a link to the obesity epidemic?. Mol Cell Endocrinol..

[r11] Engel SM, Miodovnik A, Canfield RL, Zhu C, Silva MJ, Calafat AM (2010). Prenatal phthalate exposure is associated with childhood behavior and executive functioning.. Environ Health Perspect.

[r12] Fleiss JL, Shrout PE (1977). The effects of measurement errors on some multivariate procedures.. Am J Public Health.

[r13] Frederiksen H, Skakkebaek NE, Andersson A-M (2007). Metabolism of phthalates in humans.. Mol Nutr Food Res.

[r14] Freedman DS, Khan LK, Serdula MK, Ogden CL, Dietz WH (2006). Racial and ethnic differences in secular trends for childhood BMI, weight, and height.. Obesity (Silver Spring).

[r15] Fromme H, Gruber L, Schlummer M, Wolz G, Böhmer S, Angerer J (2007). Intake of phthalates and di(2-ethylhexyl)adipate: Results of the integrated exposure assessment survey based on duplicate diet samples and biomonitoring data.. Environ Int.

[r16] Fuller WA (1987).

[r17] Grandjean P, Weihe P, White RF, Debes F, Araki S, Yokoyama K (1997). Cognitive deficit in 7-year-old children with prenatal exposure to methylmercury.. Neurotoxicol Teratol.

[r18] Grummer-Strawn LM, Reinold C, Krebs NF (2010). Use of World Health Organization and CDC growth charts for children aged 0–59 months in the United States.. MMWR Recomm Rep.

[r19] Grün F, Blumberg B (2007). Perturbed nuclear receptor signaling by environmental obesogens as emerging factors in the obesity crisis.. Rev Endocr Metab Disord.

[r20] Hatch E Nelson JW Qureshi MM Weinberg J Moore LL Singer M 2008 Association of urinary phthalate metabolite concentrations with body mass index and waist circumference: a cross-sectional study of nhanes data, 1999–2002. Environ Health 7 27; doi:10.1186/1476-069X-7-27 [Online 3 June 2008] 18522739 PMC2440739

[r21] Hauser R, Calafat AM (2005). Phthalates and human health.. Occup Environ Med.

[r22] Hauser R, Meeker JD, Park S, Silva MJ, Calafat AM (2004). Temporal variability of urinary phthalate metabolite levels in men of reproductive age.. Environ Health Perspect.

[r23] Hoppin JA, Brock JW, Davis BJ, Baird DD (2002). Reproducibility of urinary phthalate metabolites in first morning urine samples.. Environ Health Perspect.

[r24] Jones MP (1996). Indicator and stratification methods for missing explanatory variables in multiple linear regression.. J Am Statist Assoc.

[r25] Kuczmarski RJ Ogden CL Guo SS Grummer-Strawn LM Flegal KM Mei Z 2002 2000 CDC growth charts for the United States: methods and development. National Center for Health Statistics. Vital Health Stat 11(246 1 190 12043359

[r26] Lanphear BP, Dietrich K, Auinger P, Cox C (2000). Cognitive deficits associated with blood lead concentrations < 10 µg/dl in US children and adolescents.. Public Health Rep.

[r27] Lohman T, Roche A, Martore R (1998). Anthropometric Standardization Reference Manual.

[r28] Mes J, Coffin DE, Campbell DS (1974). Di-*n*-butyl-and di-2-ethylhexyl phthalate in human adipose tissue.. Bull Environ Contam Toxicol.

[r29] Miodovnik A, Engel SM, Zhu C, Ye X, Soorya LV, Silva MJ (2011). Endocrine disruptors and childhood social impairment.. Neurotoxicology.

[r30] Ogden CL, Carroll MD, Kit BK, Flegal KM (2012). Prevalence of obesity and trends in body mass index among US children and adolescents, 1999–2010.. JAMA.

[r31] Ogden CL, Kuczmarski RJ, Flegal KM, Mei Z, Guo S, Wei R (2002). Centers for Disease Control and Prevention 2000 growth charts for the United States: improvements to the 1977 National Center for Health Statistics version.. Pediatrics.

[r32] Romero-Franco M, Hernández-Ramírez RU, Calafat AM, Cebrián ME, Needham LL, Teitelbaum S (2011). Personal care product use and urinary levels of phthalate metabolites in Mexican women.. Environ Int.

[r33] Sathyanarayana S (2008). Phthalates and children’s health.. Curr Probl Pediatr Adolesc Health Care.

[r34] Sathyanarayana S, Karr CJ, Lozano P, Brown E, Calafat AM, Liu F (2008). Baby care products: Possible sources of infant phthalate exposure.. Pediatrics.

[r35] Schettler T (2006). Human exposure to phthalates via consumer products.. Int J Androl.

[r36] Silva MJ, Barr DB, Reidy JA, Malek NA, Hodge CC, Caudill SP (2004). Urinary levels of seven phthalate metabolites in the U.S. Population from the National Health and Nutrition Examination Survey (NHANES) 1999–2000.. Environ Health Perspect.

[r37] Stahlhut RW, van Wijngaarden E, Dye TD, Cook S, Swan SH (2007). Concentrations of urinary phthalate metabolites are associated with increased waist circumference and insulin resistance in adult U.S. males.. Environ Health Perspect.

[r38] Stahlhut RW, Welshons WV, Swan SH (2009). Bisphenol A data in NHANES suggest longer than expected half-life, substantial nonfood exposure, or both.. Environ Health Perspect.

[r39] Strauss RS, Pollack HA (2001). Epidemic increase in childhood overweight, 1986–1998.. JAMA.

[r40] Swan SH (2008). Environmental phthalate exposure in relation to reproductive outcomes and other health endpoints in humans.. Environ Res.

[r41] Swan SH, Main KM, Liu F, Stewart SL, Kruse RL, Calafat AM (2005). Decrease in anogenital distance among male infants with prenatal phthalate exposure.. Environ Health Perspect.

[r42] Teitelbaum SL, Mervish N, Moshier EL, Vangeepuram N, Galvez MP, Calafat AM (2012). Associations between phthalate metabolite urinary concentrations and body size measures in New York City children.. Environ Res.

[r43] Trasande L, Attina TM, Blustein J (2012). Association between urinary bisphenol A concentration and obesity prevalence in children and adolescents.. JAMA.

[r44] USDA (U.S. Department of Agriculture) (2010). Dietary Guidelines for Americans.. http://health.gov/dietaryguidelines/dga2010/dietaryguidelines2010.pdf.

[r45] Weitzman M, Cook S, Auinger P, Florin TA, Daniels S, Nguyen M (2005). Tobacco smoke exposure is associated with the metabolic syndrome in adolescents.. Circulation.

[r46] Wolff MS, Teitelbaum SL, Windham G, Pinney SM, Britton JA, Chelimo C (2007). Pilot study of urinary biomarkers of phytoestrogens, phthalates, and phenols in girls.. Environ Health Perspect.

[r47] Wormuth M, Scheringer M, Vollenweider M, Hungerbühler K (2006). What are the sources of exposure to eight frequently used phthalic acid esters in Europeans?. Risk Anal.

